# Q&A: First-hand perspectives as a member of the *BMC Medicine* Editorial Board scheme: an interview with Matthew Quaife and Shuai Li

**DOI:** 10.1186/s12916-021-02102-5

**Published:** 2021-10-04

**Authors:** Matthew Quaife, Shuai Li

**Affiliations:** 1 Centre for Mathematical Modelling of Infectious Diseases and TB Modelling Group, Department of Infectious Disease Epidemiology, Faculty of Epidemiology and Population Health, London School of Hygiene and Tropical Medicine, Keppel Street, London, WC1E 7HT UK; 2 Centre for Epidemiology and Biostatistics, Melbourne School of Population and Global Health, The University of Melbourne, 207 Bouverie Street, Carlton, Melbourne, Victoria 3053 Australia; 3 Centre for Cancer Genetic Epidemiology, Department of Public Health and Primary Care, University of Cambridge, Cambridge, UK; 4 Precision Medicine, School of Clinical Sciences at Monash Health, Monash University, Melbourne, Australia

## Introduction

Matthew Quaife is an assistant professor at the London School of Hygiene and Tropical Medicine (LSHTM), UK. His interests are health economics and mathematical modelling specialising in public health and infectious disease prevention. Currently, his research projects are focused on COVID-19 transmission in low- and middle-income settings, as well as evaluation of tuberculosis (TB) modelling to study the cost-effectiveness and equity impact of vaccines and digital adherence technologies.

Shuai Li is a Victorian Cancer Agency Early Career Research Fellow at the Centre for Epidemiology and Biostatistics at the University of Melbourne, Australia. His research interests are on genetic and epigenetic epidemiology of cancers, twin and family research, and cancer risk modelling. His main research projects are on developments of comprehensive risk models for breast cancer, investigations on the relationships of DNA methylation and health-related exposures, and applications of twin and family designs for causation assessment.

Drs. Quaife and Li are both recent members of the editorial board scheme for *BMC Medicine*, which involves working closely with our in-house editors to ensure that all manuscripts are subject to the same editorial standards and journal policies. Editorial Board Members (EBMs) handle manuscripts within their areas of expertise and oversee aspects of the peer review process from submission to acceptance.

### What is the most rewarding experience working as an EBM for *BMC Medicine*?

*MQ*: I really believe in the journal’s mission to make high quality research available open access. I previously had really good experiences with the journal as an author, both successful and unsuccessful - though the latter was still productive. I find it quite rewarding trying to contribute to the same robust but professional and friendly communication with authors about their manuscripts, and it’s incredibly satisfying when a paper you have managed through the system is finally published.

*SL*: I think the most rewarding experience is that I get a deeper understanding the editorial process of the submitted manuscripts. A manuscript needs to fit the scope and pass the threshold in order to sent out for external review – the first step for getting published. It was interesting to learn about this process.

### Did you find anything surprising or unexpected when learning about the editorial process?

*MQ*: I was previously on the editorial board of a different journal which published slightly less influential work, so the switch to thinking through whether submissions are of excellent scientific quality and policy relevance has been an interesting one. Not just assessing if manuscripts merit publication *somewhere,* but publication *here.* Otherwise all surprises have been very quickly mitigated by the excellent support we receive from the rest of the editorial team.

*SL*: I was impressed by the scope and threshold of the journal. The journal covers all topics related to medicine and has a very inclusive topic selection. The editorial team knows well about the research field of the manuscripts I handled and similar publications, so they can always give critical assessments to the submissions. They have a high threshold on the quality of papers to be published.

### What challenges did you find with handling a manuscript through peer review?

*MQ*: Finding reviewers! Particularly during the pandemic, it’s been such a busy time for infectious disease modellers and economists that when an interesting – COVID-19 or non-COVID-19 – paper appears, it has proven quite tricky to find people with enough time to review to the right level.

*SL*: I find the most challenging thing is to find suitable reviewers quickly. I am familiar with the research field of the manuscripts I handled, so for some manuscripts I know who would give a good review; however, the potential reviewers that come to mind initially may have conflicts of interest with the authors, or are too busy to review. Sometimes I need to invite many reviewers with the help of the editorial team to get enough agreed reviewers, and sometimes the process takes a long time.

### How do you find managing manuscripts that are sometimes outside of your research focus?

*MQ*: Initially I found this quite daunting considering papers which had a different disease focus from my work, or using methods slightly outside of those I use on a daily basis. Here I have benefitted from input from my editorial colleagues who are able to give initial opinions based on similar papers which have come across BMC Medicine’s desk, or have been published elsewhere. We also have a wonderful network of peer reviewers, who give incredibly valuable and insightful comments on manuscripts. If in doubt, I am always inclined to reach out to reviewers for their input.

*SL*: Fortunately, the manuscripts I have handled are all within my research focus, so I have not had the experience mentioned in the question yet. However, if that happens in the future, I think I will firstly read some publications to get myself familiar with relevant research field, and try to manage the manuscripts with the help of the editorial team. If I feel it’s not easy to do this, I would feel comfortable to discuss this with the editorial team, and ask them to find a more suitable member to handle.

### How does understanding the editorial process help you as a researcher?

*MQ*: It’s been really valuable seeing the other side of the process. The things which editors look for in a potentially influential manuscript aren’t necessarily the things that I was focusing on when writing papers, and I have really learnt the importance of a good abstract! No longer is it the thing I write last, just before pressing submit…

*SL*: I think I have learned two major things. The first thing is that the in-house editorial team has their own threshold for the manuscripts they are interested in publishing. The threshold depends on many factors, such as the quality and topic of the study, and the potential impact of the study. The second thing is that the peer review process can sometimes take very long time, especially when there are delays in finding suitable reviewers and reviewers returning their comments. The editors are unwilling to see this delay as well, and they are working hard and try their best to speed up. So as a researcher understanding this process, and understanding the issues in sending across timely and helpful reviews to the authors has been helpful.

## Data Availability

Not applicable

